# Critical STEM Literacy and the COVID-19 Pandemic

**DOI:** 10.1007/s42330-021-00150-w

**Published:** 2021-05-08

**Authors:** Martin Braund

**Affiliations:** Nelson Mandela University, South Campus, University Way, Summerstrand, Port Elizabeth, 6011 South Africa

**Keywords:** COVID-19, Mathematical and scientific literacy, Critical thinking

## Abstract

The COVID-19 pandemic has resulted in unprecedented amounts of information communicated to the public relating to STEM. The pandemic can be seen as a ‘wicked problem’ defined by high complexity, uncertainty and contested social values requiring a transdisciplinary approach formulating social policy. This article argues that a ‘Critical STEM Literacy’ is required to engage sufficiently with STEM knowledge and how science operates and informs personal health decisions. STEM literacy is necessary to critique government social policy decisions that set rules for behaviour to limit the spread of COVID-19. Ideas of scientific, mathematical and critical literacy are discussed before reviewing some current knowledge of the SARS-CoV-2 virus to aid interpretation of the examples provided. The article draws on experience of the pandemic in the United Kingdom (UK), particularly mathematical modelling used to calculate the reproductive rate (R) of COVID-19, communication of mortality and case data using graphs and the mitigation strategies of social distancing and mask wearing. In all these examples, there is an interaction of STEM with a political milieu that often misrepresents science as activity to generate one dependable truth, rather than through careful empirical validation of new knowledge. Critical STEM literacy thus requires appreciation of the social practices of science such as peer review and assessment of bias. Implications of the pandemic for STEM education in schools requiring critical thinking and in understanding disease epidemiology in a global context are discussed.

## Résumé

En raison de la pandémie causée par la COVID-19, une quantité d’information sans précédent a été communiquée au public concernant les STIM. La pandémie peut être perçue comme un « problème pernicieux» caractérisé par un niveau de complexité élevé, de l’incertitude et des valeurs sociales contestées ce qui demande une politique sociale fondée sur une approche transdisciplinaire. La thèse exposée dans le présent article est qu’une « compétence dans les STIM pour favoriser la pensée critique» est requise afin de pouvoir s’engager avec suffisamment de connaissances dans les STIM, comprendre comment agit la science et apprécier combien elle éclaire les décisions personnelles relatives à la santé. La compétence dans les STIM est nécessaire pour avoir un regard critique sur les décisions de politique sociale du gouvernement servant à établir les règles de comportement qui visent à contenir la propagation de la COVID-19. Afin d’aider dans l’interprétation des exemples présentés, des idées sur la compétence en sciences, en mathématiques et sur l’habileté à critiquer sont avancées avant l’examen de ce que l’on connait du coronavirus SRAS-CoV-2. L’article s’appuie sur l’expérience de la pandémie vécue au Royaume-Uni (R.-U.), particulièrement en ce qui concerne la modélisation mathématique utilisée pour calculer le taux de reproduction (Rt) de la COVID-19, la communication du nombre de mortalités et de données sur les cas par le biais de graphiques ainsi que les stratégies d’atténuation par la distanciation sociale et le port du masque. Dans tous ces exemples, les STIM interagissent avec un milieu politique qui représente souvent faussement la science comme une activité destinée à n’engendrer qu’une seule vérité fiable plutôt qu’à travers un processus de validation minutieuse et empirique des nouvelles connaissances. La compétence dans les STIM pour favoriser la pensée critique exige donc une compréhension des pratiques sociales qui s’appliquent à la science telles que l’examen par les pairs et l’évaluation de biais. L’on aborde les conséquences de la pandémie sur l’enseignement scolaire des STIM qui exigent une pensée critique et la compréhension de l’épidémiologie des maladies dans un contexte international.

To say the COVID-19 pandemic has affected our lives like nothing before is fast becoming a cliché. But one thing is certain; that people in every country have been exposed to unprecedented amounts of scientific, mathematical, statistical and technical information. It seems that overnight, we were confronted with concepts in virology, immunology and epidemiology and bombarded by numbers and equations from mathematical modelling and statistics and graphical representations of varying kinds and complexity. Thus, the COVID-19 pandemic can be seen as requiring knowledge and solutions from a wide range of disciplines including those concerned with STEM—Science, Technology Engineering and Mathematics—of concern to educators. As countries grappled with the spread of a disease agent science knew little about, posing a deadly threat to life and health systems and an existential one for economies, political leaders turned to (and sometimes abused or ignored) experts, modellers, statisticians and psychologists to communicate STEM information to modify human behaviour in the hope of controlling the virus. The relative successes with which the public responded to advice based on information has been key to handling the pandemic. To bring this about requires public trust that derives from sometimes problematic interactions of STEM with politics and the extent to which the public understand the nature of these interactions, the STEM concepts that underpin and are used to communicate them and how STEM, particularly science, operates in a social space where new knowledge is rapidly acquired and applied. There are two imperatives that emerge; first, that there is sufficient STEM literacy to negotiate the complex COVID-19 information landscape to enable personal decision taking and second, that this is accompanied by a degree of criticality so that politicians and experts are called to account.

There are two important caveats in presenting this article. The first is that STEM information is bounded by what we currently (in July 2020) know of SARS-CoV-2, its epidemiology and the efficacy of technological and behavioural actions to control its spread. The second is that the paper is framed mainly by the United Kingdom (UK) context for COVID-19 and ways in which the crisis has been addressed, as that is where the author has experienced the pandemic.

COVID-19, the disease characterised as a global pandemic by the World Health Organisation (WHO), is caused by the SARS-CoV-2 virus. This novel zoonotic coronavirus, discovered in 2019 in China and previously unidentified in humans, infects the respiratory tract resulting in a syndrome, which in severe cases, can be fatal. SARS-CoV-2 can trigger an excessive immune response known as a cytokine storm, leading to possible multiple organ failure and death. Damage to the kidney, liver and spleen observed in some patients with COVID-19 suggests the virus can be carried in the blood and infect various organs or tissues (Guan et al., 2020) though it is not yet clear whether more damage is done here by the cytokine storm or the virus itself.

The UK has been particularly affected by COVID-19. In the 16 weeks between 7th March and 26th June 2020, there were 49,607 deaths in England and Wales where COVID-19 was mentioned on the death certificate (ONS, 2020). In July 2020, the UK had the highest world death toll relative to population from COVID 19 at 603 per million population, ahead of the USA (424 per million), Brazil, (373 per million) and Canada (234 per million) (ONS, 2020). The severity of COVID-19 varies with age and gender. Data from the UK show that males are roughly twice as likely to die from COVID-19 and, while some young and middle-aged adults can develop serious complications or die from COVID-19, the risks rise sharply with age mainly because immune systems tend to deteriorate and because older people are more likely to have chronic underlying conditions such as respiratory and cardiac disease and diabetes or have compromised immune systems. For example, the mortality rate from COVID-19 in England and Wales at ages 80–84 is about eight times greater than at ages 60–64 and about 50 times higher than at ages 17–25 (Kings Fund, 2020; Mallapaty, 2020; ONS, 2020).

The virus can easily shed particles into saliva making a person infectious even if they show no symptoms, which did not feature in the case of previous pandemics of coronavirus SARS or MERS, where infectivity did not occur until symptoms were full-blown (Cyranoski, 2020). Transmission of SARS-CoV-2 and subsequent infection can occur by direct contact with infected individuals or by touch, though most is thought to be via respiratory droplets, of 500 nm diameter or greater, or in smaller aerosol particles less than 500 nm diameter. Respiratory droplets, being heavier, tend to drop out under gravity within 2 m but aerosol particles can travel substantial distances. Both types of particles are airborne and so transmission distances are subject to vagaries of air currents and wind (Bar-On et al., 2020; Jayaweera et al., 2020).

This article is a non-empirical, polemic arguing for a critical STEM literacy, brought into sharper focus by the requirements of the pandemic for people in all situations to engage with STEM issues, knowledge and information so that personal health decisions are informed rather than relying on hearsay, myth and false information. A recent survey in the UK showed that 42% of people obtained COVID-19 information from social media with only 22% accessing official local and government sources and even fewer (16%) using health professionals (Boyd, 2020). Though the argument for a critical STEM literacy extends beyond COVID-19, the pandemic has created a prescient need for critical STEM literacy at all levels of education.

Technology and engineering as STEM examples relating to COVID-19 of particular relevance to education include technology associated with design of PPE (personal protective equipment) and information systems involved in the track, trace and isolate actions and engineering in the repurposing of plant to manufacture PPE. For the purposes of this article, however, examples from the UK experience of the pandemic are limited to the mathematical modelling of the COVID-19 outbreak and spread, graphical representation of some statistical data and the technological and epidemiological evidence behind the wearing of face masks or coverings and social distancing to control viral spread. Before tackling these examples, in arguing for critical STEM literacy, there are sections reviewing some of the literature on public understanding and STEM literacy relevant to these examples and the wider issues surrounding COVID-19. These sections are followed by a short review of what is meant and implied by critical literacy, applied mostly to science but expanded to embrace STEM as a whole.

## STEM Literacy and Public Understanding

The body of literature potentially relevant to this article is vast, from research in scientific and mathematical understanding of COVID-19-related concepts and processes, through STEM literacy and public understanding of risk and aspects of public behaviour. To support themes of this article, there is a necessary selection of relevant work and ideas mainly in science and mathematics connected with the examples that follow and to establish the concept of critical STEM literacy.

At the beginning of the last century, Dewey advocated understanding of science for the general public, claiming that “contemporary civilization rests so largely upon applied science that no one can really understand it who does not grasp something of the scientific methods and results that underlie it…” (Dewey, 1909, p. 291). As the century advanced, with rapid technological development in many countries, the idea of ‘scientific literacy’ emerged to embrace knowledge of basic scientific facts, experimental science and how science might benefit society (Bauer, 2009, p. 223). Thus, throughout the literature, there is a tendency to use the terms literacy and Public Understanding of Science (PUS) interchangeably, sometimes with confusing overlaps.

In the 1970s and 1980s, studies of scientific literacy tended to focus on what people might or might not know. For example, Durant, Thomas and Evan’s seminal article in *Nature* (Durant et al., 1989) revealed, among many other things, that about 30% of adults in both Britain and the USA failed to realise that antibiotics are ineffective against viruses. According to a survey in the UK carried out 25 years later, it seems that this misconception is even more prevalent. The YouGov survey of 2014 found that 41% of the population surveyed believed that antibiotics kill viruses (YouGov, 2014). The dominant research paradigm in the 1980s and 1990s for science literacy, as far as the media of the time was concerned, was of knowledge deficit. Yet, less publicised aspects of Durant’s research explored public attitudes and interest in science. Of relevance to this article is Durant’s finding that knowledge of medical advances was a priority to learn about on both sides of the Atlantic; yet, those who expressed most interest did not admit to much associated knowledge (Durant et al., 1989, p. 11). In a similar vein, going beyond mere deficit aspects and in responding to Durant and others’ surveys, Miller proposed a ‘functional’ scientific literacy, being sufficient knowledge and understanding to read, engage with and debate issues published in newspapers (Miller, 1998). More recently, Feinstein (2011) argued for a science education that helps people solve personally meaningful, everyday problems and make important science-related decisions. Miller refers to a need for educating ‘competent outsiders’ able to access science relevant to their lives rather than ‘competent insiders’, sufficiently educated in science but lacking confidence to engage with and question the science they experience in everyday life.

In a similar way for science, the concept of ‘mathematical literacy’ has expanded and evolved from the more limited confines of ‘numeracy’, concerned with processes of number manipulation, to embrace a “quantitative literacy” recognising that societies “…keep increasing the use of numbers” (Jablonka, 2003, p. 77). Thus, mathematical literacy has come, like science, to be seen as ‘functional’, using mathematics to take action and decisions that impact everyday life. Consequently, the mathematics curriculum in many countries, including the UK, aims to become more contextualised. Yet, a drive for contextualisation is not necessarily linked with high performance in mathematical problem solving. As part of the OECD PISA framework for 2012, 15-year-olds were asked how often they encountered mathematics applied to real-world problems. Correlations of these data with performance on mathematical tasks showed those who *frequently* encounter applied problems scored about ten PISA score points *below* students who *sometimes* encounter such problems (OECD, 2014). It could be that this negative link between context and performance is connected with a trend for top scoring nations in mathematics assessments, such as China and in South East Asia, to concentrate on traditional decontextualised mathematical learning at the expense of context-based approaches.

Of particular relevance for this article is literature on the interpretation of graphical information prevalent in communicating about COVID-19. Graphical displays contain a multitude of information conveyed by the title, labels and axes and features of the display (e.g. size, spacing, patterns in the data) that vary in complexity. Reviewing research on graphical interpretation, Friel et al. (2001) identify three component processes: (a) to read information directly from a graph, (b) to manipulate information read from a graph and (c) to generalise, predict or identify trends. Taking this a little further, Glazer (2011) proposes that it is also necessary to read between and beyond data to usefully interact with graphs in news reports (Glazer, 2011, p. 190). A common cognitive error in graph reading is interpreting data iconically, implying ‘reading the graph as a picture’. This occurs when students view graphs as representing literal pictures of situations rather than abstract quantitative information. Other common difficulties at deeper levels include confusing the slope and the height, an interval with a point, conceiving a graph as constructed of discrete points and focussing on x–y trends all of which are added to by the total volume of information conveyed (Glazer, 2011, p. 185).

Graph comprehension is not just affected by the visual characteristics of graphs outlined above but also by the viewers’ prior knowledge and expertise in associated skills such as graphical, explanatory and reasoning skills (Shah et al., 2005). These authors warn that acceptance of a particular theory or assumed familiarity with data can lead people to see trends in data that are not really there. It seems our established views and information overload including that communicated graphically can make us ‘data blind’. This has been a prescient danger in the use of data streams in coronavirus government and media updates, especially in the UK, as we shall see later.

### Critical STEM Literacy

The idea of science literacy requiring some ability to reflect as part of wider citizenship is embodied in a statement by the OECD (Organisation for Economic Cooperation and Development) in their framework document for the 2015 PISA assessments where they define science literacy as “… the ability to engage with science related issues, and with the ideas of science, as a reflective citizen” (OECD, 2017:22). The specific term ‘Critical Science Literacy’ (CSL) has been used by Priest (2013) to expand the idea of science literacy and PUS in the modern information age, where the skills to navigate and make sense of information are crucial in selecting which truths to rely on from a list of competing claims. In this article, I expand Priest’s idea to embrace all of STEM. In Priest’s view of critical literacy, there is recognition that information technology allows people access to a mass of information from a multitude of sources but at the same time not necessarily having sufficient information literacy to assess which are reliable (Savolainen, 2007). In a recent report to the European Commission on the modern challenges of scientific literacy for educational systems, Siarova et al. (2019, p. 8) recommend education should promote effective tools to detect, analyse and expose misinformation and disinformation.

Critical science literacy requires more than knowledge of just some science background and of methods to collect evidence and validate claims, requiring knowledge of what Priest calls, the *social practices of science* (Priest, 2013, p. 139). To interact in productive ways that track through the huge amount of information available (particularly for COVID-19) means understanding at least something of how science, and the experts who communicate it, operate in a socio-political domain through these social practices. Priest points out components of social practices as: what knowledgeable practitioners might do when they encounter new claims; examining the source and its credibility in terms of expert pedigree, assessing the extent and quality of peer review, looking at relations and references to key sources and whether political or funding affiliations might lead to bias (Priest, 2013, p. 140). However, for such a public arena as COVID-19, an extensive evaluation of evidence on which claims are being made from the world of these social practices is problematic for the lay public and even for many journalists. Thus, other psycho-sociological methods or ‘heuristic cues’ (see Priest 2013, p. 139) must be used to judge the validity of claims. Priest sees CSL keeping an eye out for the exceptions to the general assumption that most honourable scientists are trustworthy and operate in ethical ways.

Until the advent of the Internet and the mass access to information it provides, a few journalists with some science knowledge and reporting expertise mediated much STEM information for public consumption. With instant access for anyone to read whatever views they wish, judging and comparing claims has become a more complex and risky business. There is a responsibility for citizens to recognise that media often represent contrarian views from outside and inside STEM circles and to recognise that so-called experts are not always of full agreement with a position, “Open-mindedness and the idea that truth is subject to revision in the light of evidence are—quite legitimately—part of the scientific landscape—and recognizing this is also part of critical science literacy” (Priest, 2013, p. 143).

The need to critically examine how science and wider STEM practices operate in the COVID-19 context are shown in relation to the examples that follow and developed further in the discussion section.

## Mathematical Literacy and COVID-19

### Mathematical Modelling and the ‘R’ number

Mathematical models designed to show spread of the virus within populations have been used by most countries to guide policy decisions in handling the COVID-19 crisis. These models are always approximations of reality and only behave with sufficient predictive power according to the quality of data fed into them and assumptions on which they are based. For example, an early model devised by Imperial College in London, based on data inputs from previous non-COVID epidemics, predicted COVID-19 would be no worse, in terms of mortality, than annual influenza. Imperial’s model was quickly updated entering new data emerging from the pandemic to show that if government took no action COVID spread could result in 500,000 deaths (Adams, 2020). Imperial’s updated modelling forced the UK government to impose a near total lockdown, though only after a period of vacillation.

Models tracking the state and progress of viral infections are based on trying to understand how quickly people move between three main states. Individuals are either susceptible to the virus, have become infected and then either recover or die (Adams, 2020). Central to all models is the idea of the reproductivity of the virus, known as the *R* rate or number. The *R* number has featured in most daily televised statements given by the UK government from March until early July. *R* (more precisely *R*o when the infection is beginning and everyone is susceptible and *R*e when the infection is spreading) gives some indication of how fast the virus might spread—the key value of one or less indicating that less than one person will be infected by another and that the virus will eventually find fewer hosts to infect and hence die out. However, the over-simplicity of reporting one number masks the sociological factors needed to derive it. In a lecture on the mathematics of COVID-19, Britton (2020) factorises *R (R*e*)* as follows:

$$R\left(e\right)=p\times c\times i$$

where (*p*) represents the transmission probability for a contact from an infected individual, (*c)* is the number of contacts per day and (*i*) is the duration of any contact.

Britton goes on to explain that *R*e is only likely to be decreased if one bears in mind all of the sociological and behavioural factors impacting (*p*), (*c*) and (*i*). For example, face mask wearing and hand washing for (*p*); quarantine, avoidance of public transport and public events for (*c*) and effective diagnosis/testing and isolation strategies for (*i*). Recently (July 2020), the value of citing the *R* number has been called into question by a group of scientists outside the UK government advisory group who claim the number is too crude and variable to determine regional variations in infection rates guiding local lockdowns (Rossman, 2020). In the resurgence of infections at local level in the UK, data on the number of COVID-19 cases per 100,000 in a population have been used rather than the *R* number to guide decisions limiting the spread of the virus. For the public and experts alike, having some idea of what lies behind numbers is important if public policy is to be trusted and effective. Just throwing one number into the mix of public data communication without much explanation does little to maintain confidence or to justify why that number is meaningful in the first place, thus warranting some mathematical criticality.

### Graphical Information

From the beginning of measures in late March to control the spread of COVID-19 in the UK, there have been daily televised briefings carried out by a member of government flanked on each side by a scientific and medical adviser. These briefings have included graphs of data on daily cases of COVID-19 from swab tests, daily deaths from COVID-19 in hospitals (and later including in care homes and the community), recovery rates, hospital admissions from COVID-19, occupancy of intensive care units (ICU) beds, numbers of patients on ventilators, international comparisons of death rates, number of swab tests carried out and transport use. These data are communicated through mixtures of bar graphs (typically for daily data on deaths and cases) and line graphs (typically for trends or rolling averages in daily data over time and international comparisons). Given the problems that people have in interpreting information from graphs reviewed earlier, one might wonder what, if anything, most people make of all this. It has often been hard even for me, as a relatively literate consumer and commentator, to cope with this much data and its display in often confusing styles and formats. The situation has not been helped by ways in which data communication via graphs has shifted in type.

**Fig. 1:**
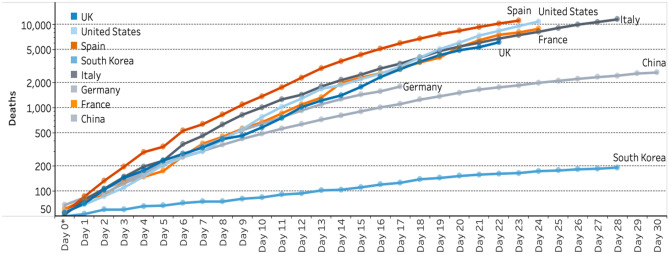
Global death comparisons for selected countries shown at the UK government’s daily COVID-19 briefing on 7 April 2020 used with permission. Her Majesty’s Government. Available under Open Government Licence: https://www.nationalarchives.gov.uk/doc/open-government-licence/version/3/

**Fig. 2:**
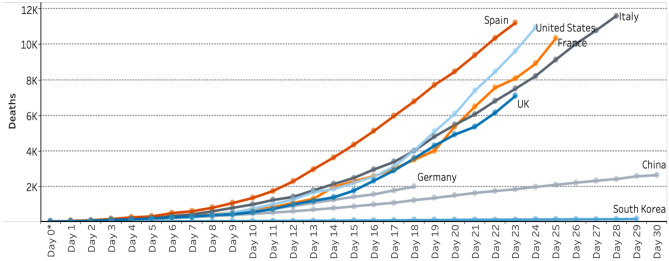
Global death comparisons for selected countries shown at the UK government’s daily COVID-19 briefing on 8th April 2020 used with permission. Her Majesty’s Government. Available under Open Government Licence: https://www.nationalarchives.gov.uk/doc/open-government-licence/version/3/

The graphs shown as Figs. 1 and 2 were used on subsequent days in COVID-19-televised briefings to give some idea of the UK position compared with a selection of countries significantly affected by COVID-19 at the time. Graphs show daily deaths since the outbreaks were first confirmed in countries, and hence, the lines are of different lengths in terms of *x*-axes. On 8th April, there was a change in graph scale from the logarithmic one used for data on 7th April (Fig. 1) to a linear scale for data shown on 8th April (Fig. 2). There appear to be three possible reasons for this. The first is that the UK and media in other countries talked up the idea of “flattening the curve,” in controlling the virus and that this trend would be easier to see in the graph in Fig. 1, using a logarithmic scale. Indeed, all data on international comparisons were shown using graphs with logarithmic scales from mid-March until the change on 8th April.

A second reason is that a logarithmic scale is much harder for people to interpret and so less likely to make them cognisant of government policy decisions. This is backed up by research by Romano et al. in the USA who found that only 40% of people shown COVID data could interpret information from a logarithmic scale compared with 84% given the same information using a linear scale (Romano et al., 2020). A third possibility is more contentious, that international comparisons and the overall picture apparent on the linear scale placed the UK in a slightly better light than on a logarithmic version, though there is no direct evidence of any intention to do this.

From early in April, death figures coming out of UK hospitals were rising at an alarming rate, but this was nothing compared to the total figures when deaths from COVID-19 in care homes and in the wider community were included. The government decision in late March to force hospitals to release beds for COVID patients by sending non-COVID long-term patients into inadequately protected care homes without testing them for COVID-19 meant there were soon more COVID-19 cases and resulting deaths in care homes than in hospitals. Eventually, the daily briefings were forced to include all deaths on graphs from the 24th April. By early May, as total COVID-19 deaths in the UK had overtaken all countries except the USA, international comparisons were dropped from briefings and graphical information given at daily briefings radically changed to include more pictograms with just a few line graphs of health information from hospitals.

**Fig. 3:**
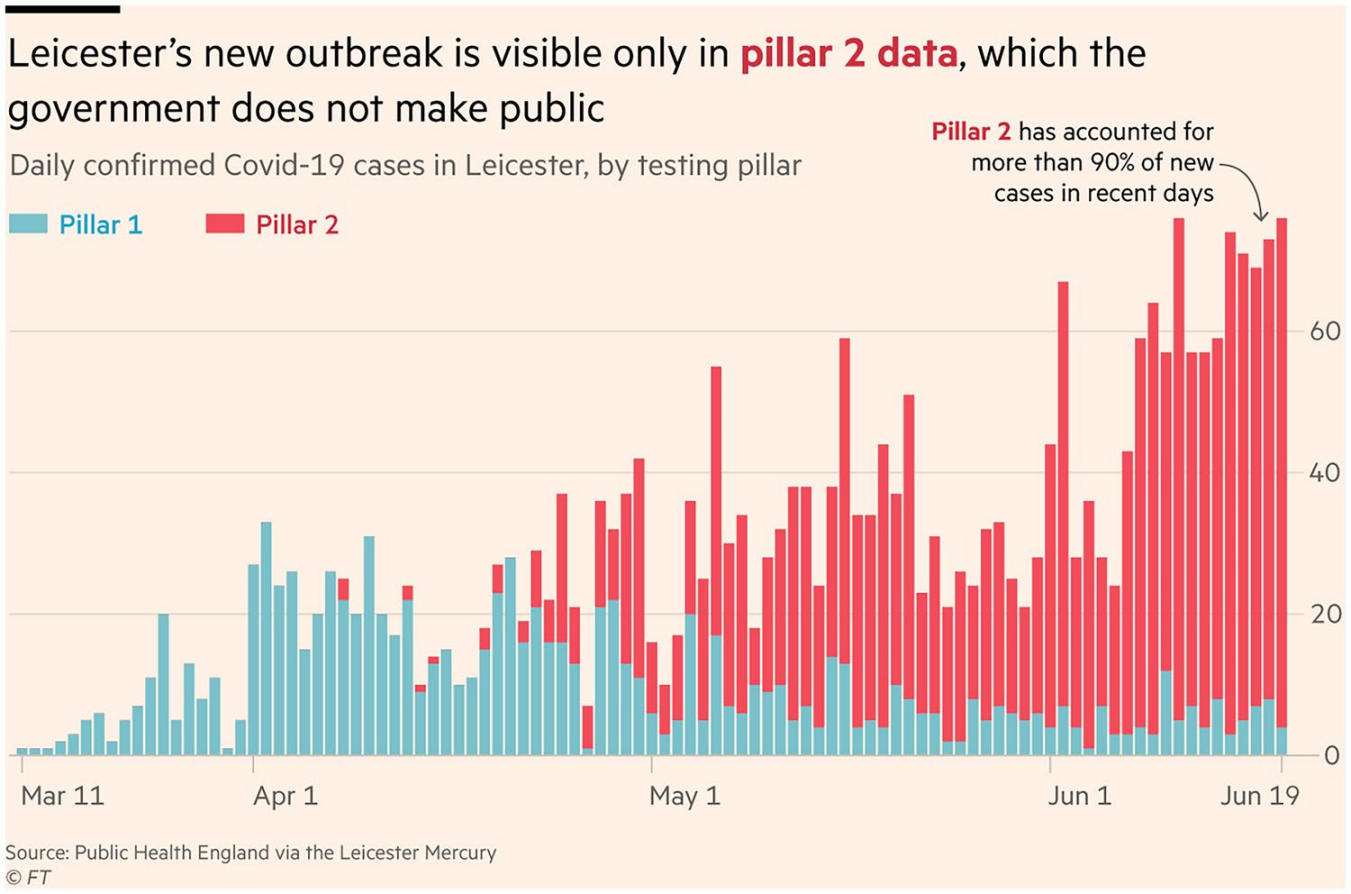
Graph showing daily test result data from COVID-19 swab tests taken in hospitals (Pillar 1 data) and in commercial and independent laboratories for the Leicester area (Pillar 2 data). (Source: John Burn-Murdoch, Sarah Neville, Laura Hughes and Andy Bounds, FT.com, 30th June 2020. Copyright FT 2020. Used under license from the Financial Times. All rights reserved.)

Later in the UK COVID-19 story, information from testing for the virus at local level was urgent to discover if and when any new outbreaks of COVID-19 should be controlled by localised lockdowns. In the graph shown as Fig. 3, daily data from COVID-19 swab tests for the city of Leicester (a city with a population of 320 K in the East Midlands of England) are shown. Daily positive results from swab tests available from hospital laboratories (Pillar 1 data in blue/light shading) seemed to show a downward and stabilising trend for numbers of cases similar to other parts of the UK at the time. But, when results from tests carried out in commercial and university laboratories (Pillar 2 data in red/darker shading) were added, an alarming surge in daily cases was evident, opposite to the trend visible for only Pillar 1 data. Thus, having complete data is necessary in situations where vital health and other decisions are made using data.

In this, and the example of international comparisons discussed earlier, there is clearly a case for critical graphical literacy enabling people to engage with crucial information and to appreciate the ways in which different graphical communication is chosen and used. For educating the public and at school level, these examples could form the basis of useful lessons and for wider critical discussion of data communication within a socio-political context.

## Mitigation Actions for COVID-19 and STEM Literacy

In most countries, suppressing the spread of SARS-CoV-2 has been a priority requiring balance of prevention and mitigation measures by persuasion or legal enforcement. Prevention includes isolation and quarantine of infected or at risk individuals and cancellation of mass crowd events, while mitigation involves strategies such as social distancing or wearing face masks to reduce the chance of infection from one person to another.

### Social Distancing

The idea of people being separated by a distance, somewhere between 1 and 2 m, is that most respiratory droplets transmitted from people that might contain SARS-CoV-2 or fragments thereof are thought to drop out under gravity or are less prevalent at a distance (SAGE, 2020; WHO, 2020). The World Health Organisation (WHO) recommended distance is 1 m followed by China, Denmark, France, Hong Kong and Singapore. India has been maintaining 2 m distance as has the UK, Canada and Switzerland. Australia, Belgium, Greece, Germany, Italy, Spain and Portugal have prescribed 1.5 m for their physical distance rule, while the USA and South Korea have opted for specific distances of 1.8 m and 1.4 m, respectively. A briefing paper produced by SAGE (Scientific Advisory Group for Emergencies) concluded, from a meta-analysis of studies on distancing, that it is not possible to say with certainty what a safe distance of separation is, but that best current evidence suggests separation of 1 m carries between 2 and 10 times the risk of infection compared with a 2 m separation (SAGE, 2020, p.  21).

A critical examination of the simple idea that social distancing keeps you safe from infection is not difficult. Vagaries of environmental conditions, air movements from ventilation and air conditioning systems and human factors such as the amount of coughing and other methods of violently exhaling respiratory particles in droplets or aerosols are all factors affecting spread of the virus (SAGE, 2020) that question distance rules. For example, the Skagit Chorale outbreak in the US state of Washington resulted in 33 confirmed and 20 probable cases of COVID-19 among 61 people from one infector in a 2.5 h period. Transmission is said to have included aerosol spread exacerbated through singing (Hamner, 2020). Findings such as this have led the UK government to be reluctant to open theatres and other venues where enhanced exhalation might feature, though the reasoning behind such decisions, as for so many COVID-19-related policy actions, is hardly ever discussed.

### Face Masks

At the time of writing (July 2020), the wearing of masks or face coverings was the most controversial aspect of government policy for mitigation of COVID-19 in the UK. Back in early April, when there was acute rise in COVID cases in the UK, wearing masks was seen as weak mitigation in the light of conflicting or inconclusive evidence emerging from reviews of research. The claim was made that evidence for efficacy of mask wearing came from limited studies of non-SARS-CoV-2 viral outbreaks or from studies that were not RCTs (randomly controlled trials). The same could have been said, however, for coughing into your elbow, social distancing and quarantine; yet, these measures were seen as effective and widely adopted (Royal Society, 2020). The situation was not helped by advice from the WHO who expressed similar caution on evidence for wearing face masks.

What seems to have shifted the UK government stance on mask wearing in early July is mounting pressure from virologists, immunologists and epidemiologists that, on balance, wearing masks could make a second wave of infection in community spread less likely. Partly, this was due to increasing evidence that aerosols containing respiratory droplets evaporated to less than 500 nm in diameter are carried substantial distances in the atmosphere where they are subsequently inhaled (He et al., 2020; Royal Society, 2020; SAGE, 2020). As seen in the Skagit Chorale outbreak, both droplet and aerosol transmission is increased by strong respiratory actions. There is also evidence that poor ventilation such as in shops, offices and factories may cause recirculation of viral laden air (He et al., 2020). An additional factor, discussed by He et al., is that viral spreading by droplets or aerosols depends on the progression of the disease and may be highest the day prior to symptom onset. This confirms the idea, promoted early in the outbreak, that people who do not know they have COVID-19 are just as likely to be infectious and spread the virus as those who do. On balance, the consensus of opinion is that while masks can prevent some ingress of viral particles, they can act most to reduce exhalation from infected individuals and, when used alongside other measures such as social distancing, would therefore make a contribution to controlling the spread of COVID-19.

The UK government had already moved to make wearing of masks compulsory in hospitals and on all public transport, but as of 24th July, extended this to all shops and enclosed public spaces, following advice from SAGE, The Royal Society, and because of the changing position of the WHO. A rapid review of evidence on transmission of SARS-CoV-2 for the Royal Society concluded:

“Cloth face coverings are effective in reducing source virus transmission, i.e., outward protection of others, when they are of optimal material and construction (high grade cotton, hybrid and multilayer) and fitted correctly and for source protection of the wearer”

(Royal Society, 2020, pre-print: 1)

Like social distancing and many other aspects of the COVID-19 pandemic, public understanding of the nature and status of STEM research and knowledge contributes to the overall trust which the population can place on decisions affecting their lives and indeed that may even save them. As the Royal Society review goes on to state:

“Socio-behavioural factors are vital to understanding public adherence to wearing face masks and coverings, including public understanding of virus transmission, risk perception, trust, altruism, individual traits, perceived barriers.”

(Royal Society, 2020, pre-print: 1)

How these aspects in the quote above interact in the socio-political milieu for STEM literacy helps frame discussion in the next section.

## Discussion

Clear communication of mathematical, graphical and scientific-epidemiological information has been essential in building trust to effect sufficient public actions controlling the spread of COVID-19. Underpinning this is public understanding at a basic level about diseases and how they are spread, how the social practices of STEM interact within the socio-political sphere and critical thinking and information literacy needed to evaluate sources of claims and their validity. A discussion of what this might mean for education systems follows some discussion of critical literacy in relation to the social practices of science/STEM.

### Critical Literacy and the Social Practices of Science/STEM

In the UK, the public have received daily televised updates on the pandemic in which the prime minister, or a cabinet substitute, state how the government is “following the science”. The notion of “THE science” gives an impression of just one (most believable) interpretation. This is to wholly misrepresent science and its social practices, which proceed on a multitude of ideas subjected through empiricism and peer review to establish which, if any, have more substance than others. In early, UK broadcasts the idea of “herd immunity” was promoted, letting immunity develop in the population thus avoiding a lockdown damaging the economy. Other ideas and models, such as ones discussed earlier from Imperial College, were available but never acknowledged. As soon as the government realised, from the revised Imperial model, that over 500,000 COVID-19 deaths could result from the herd immunity plan, they backtracked to impose a lockdown, though the delay may have cost over 20,000 excess deaths according to one of the government’s own modellers (Buchan, 2020). The government’s response to public criticism of such indecision was, perhaps, predictable that “they were only following the science”.

In these cases, it is clear there is a relationship between politicians and STEM and other ‘experts’ that the public should be educated to understand. Governments responding to the pandemic have a difficult task balancing health actions informed by STEM with wider needs of people (for example, their mental health and wellbeing) and the economy. As the deputy chief medical officer for England noted, the government’s decisions are based on a combination of “science, politics and practicality” (Neilan, 2020). In this, it is important to educate that scientists often disagree more than they agree. In the local resurgence of COVID-19 cases in England, there are key differences of opinion of medical experts as to how to handle the rapid rise in infection rates. On the one hand, a group of 30 epidemiologists and health professionals call for shielding more vulnerable groups of the population, for example those over 65 and/or with underlying medical conditions while keeping economic activity fully open. On the other hand are a group who argue to increase lockdowns of most economic and hospitality activity for the whole population in the hope of slowing spread of COVID-19 (Wise, 2020).

An understanding of the social practices of science is also required to challenge false hopes and expectations. Early in the pandemic (in March 2020), the media and some politicians alluded to a vaccine for COVID-19 being available by the end of 2020, which at the time seemed fanciful. Rarely was any information given as to what is entailed in researching, testing and trialling vaccines before they are licensed for large-scale public use or the time it takes for all stages to be completed. At the time of writing (July 2020), the UK was considering vaccines developed by six different research groups and best estimates were that a vaccine for public use would not emerge until at least March 2021 . It is to the great credit of all involved, however, that what seemed almost impossible in March 2020 was achieved, with the first vaccinations using the Pfizer-BioNTech vaccine taking place in the UK late in December 2020.

### Critical STEM Literacy and Education

Developing critical STEM literacy requires actions at systemic level in how the subject-oriented curriculum is conceived as well as what can be done at subject level.

The COVID-19 pandemic necessitated a response bringing together knowledge and expertise from disciplines that rarely collaborate so closely. To understand transmission of SARS-CoV-2 virus and control or mitigate its spread requires collaboration of virologists, immunologists, epidemiologists, medical practitioners and mathematicians with physicists, who explore the dynamics of air currents in transmissions, engineers who design PPE and repurpose engineering plant to manufacture it and technologists to design effective but cheap face masks and design information systems to track and trace infections. Add the psychologists and sociologists, who model and predict associated mental health and human behaviour trends, with economists, who predict and plan to mediate subsequent damage of the pandemic for regional, national and global economies, and you have a truly transdisciplinary example going beyond mere STEM. Educating for and about COVID-19 is an example of what Sharma (2020) calls ‘phronetic science’, responding to and helping understand ‘wicked problems’. Wicked problems are understood as social policy problems defined by high complexity, uncertainty and contested social values (Rittel & Webber, 1973). By this definition, the COVID-19 pandemic seems ‘extremely wicked’.

Education systems in many countries are ill-designed to deal with wicked problems in the way they compartmentalise knowledge. I have previously argued for a change in approach to STEM education valuing transdisciplinarity, where society and community needs set agendas for learning rather than requirements of curriculum subjects themselves (Braund, 2020). In achieving this, education, particularly that in STEM, will need to undergo a paradigm shift, from one where knowledge is received, static and unchallengeable to one where ideas and actions are engaged with in a substantial shift to critical literacy (see Roth & Barton, 2004). However, it is unrealistic to assume a transdisciplinary approach could or should replace subjects of the curriculum. The subject training of most secondary teachers, assessment structures and the fabric of progression through subject-oriented phases towards qualifications are real constraints. There are, however, initiatives that have successfully introduced transdisciplinary learning within a subject oriented curriculum. Most notable are initiatives in STEAM (Science, Technology, Engineering, Arts and Mathematics) where the idea is to work in ways avoiding artificial combinations (or separations) of subject disciplines that draw in arts and humanities learning with STEM activity (Braund, 2020; Yakman, 2010).

The COVID-19 pandemic has required actions from all sectors of the population that often impinge on personal liberties. Here, there are educational implications beyond STEM but that could be included as a social dimension. I have already discussed some of the STEM factors that make social distancing and wearing face masks problematic in terms of critical decisions at a personal level. The vagaries of viral transmission from person to person that result from seemingly innocent human actions such as shouting and singing, even talking loudly, are anathema to many people. The idea of wearing face masks seems a personal intrusion in a culture where, unlike in many Asian countries, this has never been a custom.

Mitigation at the personal level requires assessment of relative risk and here we can draw on research from previous experiences of pandemics to explain how people behave. Reviewing research on reactions to emerging infectious diseases (EIDs) such as HIV, Ebola and H5N1 (bird flu), Joffe comments that EIDs are often seen as non-threatening and low risk because they are either geographically distanced or are seen as only being dangerous to ‘others’ (Joffe, 2011, p. 452). In the case of COVID-19, rapid global spread of the disease has dispelled the first of these, but the idea of the virus only impacting others has become a major challenge for STEM advisers and governments alike. We have seen in many countries the tendency for large gatherings of people at demonstrations, celebrations and tourist hotspots such as beaches and that the age profile in these gatherings is mainly of people under 40, a lower risk age group for the most serious consequences of COVID-19. The fact that many people, especially in this age group, can be asymptomatic carriers for SARS-CoV-2 but can transmit virus to more vulnerable others seems to be missed. There is a case here for including a moral aspect to critical literacy proposed in this article, something that could form the basis for productive debates and activities in schools, colleges and youth groups both in STEM lessons and in education for citizenship and social justice, part of the curriculum of many countries.

Critical STEM literacy requires, as Priest (2013) recognised, knowledge of the social practices of science to educate a more discerning public. In a paper published in response to a recent call for COVID-related discussion for the journal Science and Education, Reiss (2020) recommends including aspects of the historical, philosophical and social background of STEM in the school curriculum to educate about the social practices of science. There have been two attempts in the UK at trying to widen science teaching to encompass these aspects and to relate science to its social practices, first at the inception of the National Curriculum in 1989 and again in the 2000s when an element called “How Science Works” was included (see, Braund & Campbell, 2010 for a discussion of this). Yet, these initiatives have been short lived and science teaching, at least in the UK, seems to have retrenched to its canonical teaching. The pandemic brings into sharper focus a need to revisit this area of the science curriculum.

The word ‘critical’ in critical STEM literacy implies supporting the values of critical thinking, not only in STEM but in the wider curriculum. According to a recent OECD report (OECD, 2019, p. 20), critical thinking aims to carefully evaluate and judge statements, ideas and theories relative to alternative explanations or solutions so as to reach a competent, independent position—possibly for action. Critical thinking needs not lead to an original position to a problem but it typically involves the examination and evaluation of different possible positions. In this, there is a way for debate and discussion to occur that we should encourage in both students in school and the wider general public. There is a note of caution here that unreasoned and unfettered criticality for its own sake might limit effective STEM literacy. Fotou and Constantinou (2020) point out dangers of science literacy promoting the idea of *nullis in verba* (take nobody’s word for it). In this, there is the assumption that a degree of criticality has gone too far—‘it is critical of everything’. Fotou and Constantinou go on to argue for a science literacy that is more holistic and encourages looking beyond personal impacts and actions to wider responsibilities towards others.

Evidence that many learners leave school with persistent misconceptions about viral infections (Durant et al., 1989; YouGov, 2014) and that at least half of European adults admit to a very low biology and health literacy impeding their health decisions (Sørensen et al., 2015), show a revaluation of the content of life sciences learning is urgently required. The COVID-19 crisis brings an overhaul of teaching into sharper focus. There is a case, as Reiss points out (Reiss, 2020, p. 4), to bring the historical study of diseases and how health crises have been handled into mainstream education. Reiss lists the content he taught that related to disease biology including looking at health issues internationally such as to combat malaria, HIV/AIDS, TB, cholera and diphtheria and other diseases which had been largely eradicated or controlled in the West such as smallpox and measles (Reiss, 2020, p. 2). I recognise every one of his examples and his list could be mine, yet I wonder how much of this biology gets taught in schools across the world today. Awareness of how global pandemics affect people in different countries in very different ways depending on the capacity and preparedness of health systems and degrees of social deprivation should be a key focus, and so one that educates about life sciences in the context of social justice.

Finally, there is an imperative to link teaching about pandemics of zoonotic origin through environmental education. The degradation of landscapes through increasing urbanisation that brings wildlife into closer contact with humans has been the focus of speculation that zoonoses, through which viruses such as SARS-CoV-2 spill over from animals to the human population, are made more likely (Bradley & Altizer, 2007; Neiderud, 2015). Debates on this issue and on exploitation of wild animals for food and health products which has also been connected with zoonotic events (Swift et al., 2007) are a priority to educate future generations and so to call for actions that reduce risks of further pandemics.

## Conclusion

In this article, I draw on the UK experience of the COVID-19 pandemic to show how an understanding in STEM, especially how STEM information and data are communicated and understood, builds a wider case for critical STEM literacy. The UK context has readily provided examples for me to comment on and critique such as changes in mathematical modelling, sometimes confusing communication of data as graphs and reasoning for mitigation strategies such as mask wearing and social distancing. Perhaps, STEM educators in countries that have been more effective than the UK has at controlling the pandemic would not find such fruitful ground to compose an article such as this, but I believe the argument for Critical STEM literacy is universal and holds true wherever you are.

I have conceived critical STEM literacy comprising STEM knowledge, skills and understanding necessary to engage with concepts and processes impacting personal health decisions, and engaging with interactions of STEM as part of a wider socio-political milieu. Thus, I do not distinguish between literacy and public understanding of science (PUS), preferring to combine them for a critical STEM literacy applied to wicked problems such as COVID-19. Priorities for how educational systems might respond to the COVID-19 pandemic include valuing transdisciplinary learning, teaching about the social and epistemological practices of science and the moral obligations of personal mitigation behaviour in dealing with disease spread. Additionally, the pandemic, caused by a zoonotic virus, reinforces a need for teaching about diseases and their origins within global and environmental contexts.

Ultimately, the COVID-19 pandemic requires STEM to offer wider engagement with what have been called social scientific issues (SSIs). As Ziman noted back in 1994, SSIs have been missing in STEM and science education, “where the sciences are presented as if they had no social context, no social influence and were of no social concern” (Ziman, 1994, p. 28). In spite of effort to make STEM education more engaging through recognition of the value of teaching in context, little has changed. In any turn to a curriculum that is globally responsive, has SSIs at its heart and is based on social justice, there has to be a more focussed sense of critical literacy to develop the habits of mind needed to address the wicked problems of the world that will surely proliferate but that future generations must try to solve.
